# Impact of Problematic Smartphone Use and Instagram Use Intensity on Self-Esteem with University Students from Physical Education

**DOI:** 10.3390/ijerph17124336

**Published:** 2020-06-17

**Authors:** José-María Romero-Rodríguez, Inmaculada Aznar-Díaz, José-Antonio Marín-Marín, Rebeca Soler-Costa, Carmen Rodríguez-Jiménez

**Affiliations:** 1 Department of Didactics and School Organization, University of Granada, 18071 Granada, Spain; romejo@ugr.es (J.-M.R.-R.); iaznar@ugr.es (I.A.-D.); carmenrj@ugr.es (C.R.-J.); 2 Department of Didactics and School Organization, University of Zaragoza, 50009 Zaragoza, Spain; rsoler@unizar.es

**Keywords:** problematic smartphone use, Instagram use intensity, Physical Education, university students, self-esteem

## Abstract

Mobile devices are a revolutionary element that offer many possibilities, although they can also cause problems for users. This is the case with the development of addictive behaviors that can affect personal well-being. The purpose of this paper has been to analyze the influence of smartphone addiction and Instagram use intensity on the self-esteem of Physical Education students. A cross-sectional research design was adopted by applying an online survey to a sample of undergraduate students (*n* = 385). The results showed that gender and age were factors that influenced the problematic use of the smartphone. In turn, there was a significant positive correlation between smartphone addiction and Instagram use intensity. The influence of smartphone addiction on students’ self-esteem was also highlighted. In contrast, Instagram use intensity did not affect self-esteem. Finally, the findings are discussed, and the main implications of the study are established, where physical education students take on a special role in order to avoid the improper use of smartphones and Instagram through sport.

## 1. Introduction

Our present-day society is, in a generalized way, centered on technology and everything derived from it. Thus, people are increasingly dependent on this element [1]. The cell phone as a revolutionary element of the world as we understand it, has also been subject to change since the advent of the Internet and the different applications requiring its use [2].

Mobile devices have managed to make the world of video games, shopping and quick and easy access to information—among many other things—accessible to any user. In the same way, the information exchange of information through platforms and social networks has been streamlined and transformed, since the Internet has been a before and after in this regard [3].

To all this, we must add the appearance of smartphones [4], which have contributed to the development of these potentialities regarding intercommunications and access to the network [5]. 

However, despite the advantages of these advances in today’s society [6], The appearance of the smartphone and, therefore, its increasing use in all areas and contexts where its users operate, has also reported an irresponsible use of them that has caused the appearance of new phenomena such as cyber bullying [7,8], sexting [9,10] or addiction to these devices [11].

Similarly, the use of mobile phones has meant a massive use of elements such as social networks. These represent at the present moment a basic virtual element that is used by the majority of people in society [12]. Undoubtedly, where these elements are most successful is among young people, since they allow relations and the exchange of information and content on any subject to be reached between different levels. [13]. 

This new scenario presents novel situations where users browse and group according to their interests, tastes, age, etc. [14]. In this context is the social network Instagram, which in spite of being only a few years old among the population enjoys great relevance and presence among young people, occupying many hours of the daily life of its users. This social network enables different options for communication and for sharing audio-visual content, such as permanently publishing photos, videos or texts, however users may choose to limit that published material to only 24 hours. All these possibilities are complemented by the location, date and time—in real time or delayed [15]. 

This application has undergone a rapid growth in recent times, being a great source from which to extract content of different kinds in which users, although of different ages, belong mostly to what is understood to be adolescents and young people [16,17,18]. 

The advantages of using this social network are varied and affect elements such as oral or linguistic competence in the mother tongue or in a foreign language [19,20,21]. However, the inappropriate and irresponsible use of the Internet and its associated tools such as Instagram can cause different disorders, among which smartphone addiction and excessive use of this social network stand out. This causes a series of consequences, extrapolated to the excessive use of other social networks, since the individual’s commitment to this extreme behavior is problematic [22,23]. These consequences are as follows [24]: Loss of control; Interpersonal problems; Loneliness; Increased stress; Waste of time; Decreased performance; and Depression.

The question here arises when discerning what is causing this, the problematic smartphone use (PSU) or the social network in particular. It can be about the technology itself what causes it or what the technology provides [25]. There are several investigations that advocate more for the line of thought that considers that what makes users addicts is what they achieve through technology or through specific social networks [26,27]. This is due to the false feeling of happiness and acceptance that applications such as Instagram generate through “likes” and positive comments on publications. 

As it has been seen, some of the consequences are directly associated with emotions and their management. A fundamental element to take into account in this regard is self-esteem, since it is likely to be affected when these phenomena occur [1]. Self-esteem should be understood as the evaluation that the person makes of himself, being aware of his limits, shortcomings and possibilities, judging his own actions and the importance of oneself [28]. Thus, there are many investigations [29,30,31,32] that clearly relate low self-esteem with a greater probability of falling into an addiction with respect to mobile phones or social networks such as Instagram [11,27,29], since it will also be associated with other elements such as insecurity in their social skills and attributes, and they need to be reaffirmed through the comments or opinions of third parties [33]. 

The youth population is the most vulnerable to this type of phenomenon, so a responsible education is necessary at this stage and throughout their training in the use not only of the devices, but of the management of their online profiles and the treatment of his information [34]. 

Due to all the aforementioned, the training environments have had to adapt for the implementation of all these elements in the Teaching-Learning (T-L) processes, creating new educational models and innovative methodologies, thus promoting the interconnection between the formal learning of the educational centers and informal learning that happens through technological resources outside the classroom [35].

Specifically, in the Spanish sphere, research in this regard in recent years has centered on different phenomena related to the Internet and its use [36,37], the problems that arise [38], and the impact of different social networks in different educational stages [39,40,41].

Based on these considerations, the objective of the study was to analyze the influence of smartphone addiction and Instagram use intensity on the self-esteem of the students of physical education. The research question was:

RQ1. What factors influence and what kind of influence is generated between the addiction to smartphones, Instagram use intensity and self-esteem of university students?

## 2. Materials and Methods

### 2.1. Participants and Procedure

A cross-sectional study design was adopted from the application of an online survey to a sample of undergraduate university students (*n* = 385). The research was conducted based on a convenience sampling design. Before answering the questionnaire, students were informed of the purpose of the investigation and the anonymous treatment of their data. The informed consent of each participant was an essential requirement to participate in the study.

Specifically, the students were enrolled in the Physical Education specialty of the Primary Education Degree from various universities in southern Spain. The sample was made up of 147 men (38.2%) and 238 women (61.8%), with ages between 18 and 35 years (M = 22.17; Standard Deviation – SD = 4.89). The distribution of the age range was less than or equal to 20 years (*n* = 100; 26%), and from 21 to 35 years (*n* = 285; 74%). Students’ employment status was divided into active (*n* = 199; 51.7%) and inactive (*n* = 186; 48.3%). The data collection period was established from January to February 2020.

### 2.2. Measures and Instruments

#### 2.2.1. Social Media Intensity Scale (SMIS)

To assess the intensity of use in social networks, the Social Media Intensity Scale (SMIS) was used [42]. Specifically, the SMIS adaptation to the Instagram social network was applied [43]. The scale measures the intensity of Instagram use based on the response to seven items, with a four-level Likert scale response mode (1 = Strongly disagree; 4 = Strongly agree). Scores range from 7 to 28 points, higher scores on the scale indicate a higher level of use, intensity and engagement with Instagram, implying problematic use of Instagram. The psychometric properties and internal consistency of the scale are good [42]. The reliability obtained in this study through the Cronbach’s alpha coefficient was adequate (α = 0.813).

#### 2.2.2. Smartphone Addiction Scale (SAS-SV)

The PSU was evaluated through the short version of the Smartphone Addiction Scale (SAS-SV) [44]. The scale evaluates smartphone addiction based on the response to 10 items, with a four-level Likert scale response mode (1 = Strongly disagree; 4 = Strongly agree). Scores range from 10 to 40 points, higher scores on the scale indicate a higher degree of smartphone addiction. The scale in its multiple applications has collected adequate psychometric properties [45,46,47]. The reliability obtained in this study through the Cronbach’s alpha coefficient was adequate (α = 0.822).

#### 2.2.3. Rosenberg’s Self-Esteem Scale (RSES)

Self-esteem was assessed with the application of the Rosenberg’s Self-Esteem Scale (RSES) [48]. The Rosenberg scale evaluates global self-esteem from 10 items, with a four-level Likert scale response mode (1 = Strongly disagree; 4 = Strongly agree). The first five items present positive statements and the remaining five negative statements. The scale scores range between 10 and 40 points, the highest scores are associated with high self-esteem. The psychometric properties and internal consistency of the RSES are adequate, it is the most widely used instrument in the evaluation of self-esteem and presents its own Spanish adaptation [49]. The reliability obtained in this study through the Cronbach’s alpha coefficient was adequate (α = 0.820).

### 2.3. Data Analysis

Different statistical tests were used; firstly the mean values and standard deviations for each independent variable were calculated and the possible existence of significant differences between groups was verified through the T test for independent samples.

To establish the structural equation model, it was necessary to confirm the hypothesis of multivariate normality of the data. The univariate normality values of each scale were previously calculated using the Kolmogorov–Smirnov test to posteriorly check the hypothesis of multivariate normality from the Mardia coefficient [50]. The model’s goodness of fit indexes was also estimated, and were adequate [51,52]. Attention was paid to measures relating to the ratio χ^2^/df; Goodness-of-Fit Index (GFI); Root Mean Squared Error of Approximation (RMSEA); Normalised Fit Index (NFI); Comparative Fit Index (CFI); Adjusted Goodness-of-Fit Index (AGFI); Standardized Root Mean Square Residual (SRMR).

After this, the hypothesis testing was carried out from the path analysis, where the relationships between endogenous and exogenous variables were established.

Data analysis was carried out with the help of the statistical packages IBM SPSS and IBM SPSS Amos, version 24 (IBM Corp., Armonk, NY, USA).

## 3. Results

The mean values obtained on the three scales were disparate depending on gender, age and employment situation (Table 1). However, significant differences were established between the age groups regarding the Instagram use intensity (*p* = 0.000), with the highest average score corresponding to the stratum of 20 years or less (*M* = 18.66), and with respect to the PSU (*p* = 0.034), where the highest average score was obtained by the stratum of 20 years or less (*M* = 20.93). Significant differences were also established between the employment status of students in the Instagram use intensity (***p*** = 0.002) and PSU (***p*** = 0.011), where the highest average score was obtained in the inactive population for both cases (Instagram use intensity ***M*** = 17.54; PSU ***M*** = 20.66). Regarding gender, no significant differences were established on any scale, as was self-esteem, which was not a factor where differences were generated between population groups.

**Figure 1:**
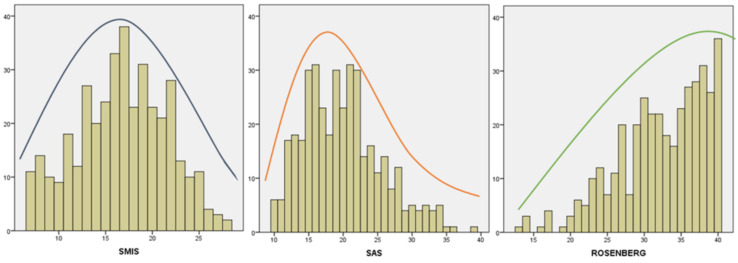
Histograms on the distribution of data by construct; SMIS = Social Media Intensity Scale; SAS = Smartphone Addiction Scale; ROSENBERG = Rosenberg’s Self-Esteem Scale.

In relation to univariate normality, the asymmetry showed a symmetric curve for the SMIS scale data, for the SAS-SV scale data the curve was asymmetrically positive and for the Rosenberg scale data the curve was established with a negative asymmetry. While the kurtosis took a platicúrtica distribution for SMIS, mesocúrtica for SAS-SV and leptocúrtica for the Rosenberg scale [53]. For its part, the Kolmogorov–Smirnov test showed that the distribution of the data was not normal (*p* < 0.05) (Table 2). Thus, it was necessary to check normality through the distribution of the scores in the histograms for each scale (Figure 1). Histograms showed normality for the SMIS scale data. The data distribution on SAS-SV and the Rosenberg scale did not follow a normal distribution.

Although there was no univariate normality in some cases, the hypothesis of multivariate normality was confirmed (Mardia = 58,743). This value was less than 783, a result of the formula p * (p + 2) [54], where p corresponded to 27 (total number of observed variables). The confirmation of multivariate normality served to obtain evidence of the adequacy of the data for the preparation of the SEM. In turn, the SEM goodness-of-fit indices were adequate based on the criteria established for each of them (Table 3). 

On the other hand, the estimates established in the path analysis collected only significant values in: the relationship between gender and Instagram use intensity (*p* = 0.048), being a positive and significant influence; age relationship and Instagram use intensity (*p* = ***), being a negative and significant influence; relationship Instagram use intensity and number of photographs (*p* = ***), being a positive and significant influence; relationship Instagram use intensity and number of followers (*p* = ***), being a positive and significant influence; PSU and self-esteem relationship (*p* = ***), being a negative and significant influence (Table 4). The correlation between Instagram use intensity and PSU was also significant and positive (*p* = ***).

**Figure 2:**
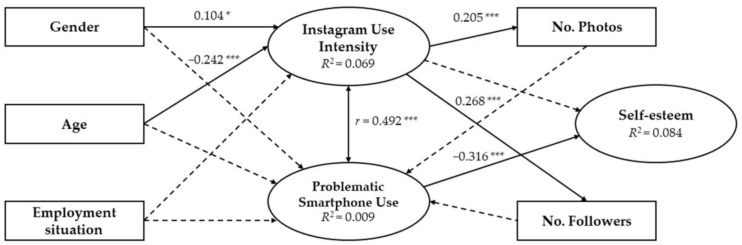
Estimates of the structural equation model. Note: *r* = correlation coefficient; *p < 0.05; ***p < 0.001. Discontinuous arrow = not significant.

The path analysis graphically collected the associations between the study variables. Only the coefficients that were significant were shown to facilitate the interpretation of the data (Figure 2). The main constructs were Instagram use intensity and PSU, which were both influenced by three independent variables (gender, age and employment situation). In the case of PSU, the influence of the number of photos and number of followers was also established. In turn, the relationship of the Instagram use intensity with the number of photos, number of followers and with self-esteem was established. The relationship between PSU and self-esteem was also shown. Finally, the correlation between the two main constructs was exemplified: Instagram use intensity and PSU. The percentage of variation of each construct established by the coefficient of determination was 6.9% for Instagram use intensity (*R^2^* = 0.069), 0.9% for PSU (*R^2^* = 0.009), and 8.4% for self-esteem. (*R*^2^ = 0.084).

## 4. Discussion

The 21st century society is characterized, among other things, by the use of technology as an inescapable instrument for communicating and relating. The appearance of mobile telephony at the end of the last century opened the door to communication beyond the limits of homes and industries to extend relationships to other areas and spaces. An exponential leap in these levels of communication occurred with the incursion of the smartphone in the lives of people. The possibilities they offered to interact and explore new forms of interaction also brought about the appearance of new problems associated with the use of these devices and the applications to which they had access [7,8,9,10,11]. In this sense, the study carried out on the problem of the use of the smartphone, the social network Instagram, and its influence on their self-esteem in university students has produced very enriching data which is in tune with other previous studies. [25,26,27].

In response to the questions that were posed at the beginning of this study, among the interesting findings is the non-existence of significant differences according to gender in the three scales (SMIS, SAS-SV and RSES) (Table 1), so it can be assumed that both smartphone addiction, intense use of social networks and self-esteem do not depend on gender. On the other hand, when the variable is age, there are significant differences, both in Instagram use intensity and in PSU, in those who are 20 years old or younger, corroborating previous studies [13].

On the other hand, and based on sociodemographic factors, specifically, with the work situation, the existence of higher scores, and with significant differences, in the Instagram use intensity and the PSU in those people in those whose work situation is inactive has been observed. This fact may be derived from the consequences already listed [23,24], which is coupled with excessive use of social networks and smartphones.

Another of the questions raised in this work was the degree of intensity of use of Instagram by university students and the influence of other sociodemographic factors. In this sense, the results show that both gender and age are two variables that contribute significantly to the intensive use of this social network, as can be seen through path analysis. Furthermore, the data suggest that Instagram use intensity significantly influenced the number of photos published on the social network, in that the influence was positive; that is, the more intensive use, the greater number of followers, and vice versa. This same prevalence occurs between the Instagram use intensity and the increase in followers on this social network. More Instagram use intensity encourages more followers, and vice versa.

A relevant aspect of this research is the correlation between Instagram use intensity and PSU. Thus, the SRW value determines that the relationship between these two variables is positive and highly significant, revealing that the more intensive use of the social network is made, the greater the addiction to the smartphone will be and vice versa. These results are clearly in line with the most recent studies [38,40,41,43].

According to the parameter estimates of the final model, the employment situation is not an element that significantly influences Instagram use intensity or PSU. Similarly, other sociodemographic factors such as age and gender or exogenous factors such as the number of photographs and the number of followers, are also not significant on the PSU. These data are important because they confirm the idea that smartphone addiction is not promoted by the device itself, but that the use of certain applications is what determines and encourages problematic use of this device [25,36]. 

Regarding self-esteem, the results obtained (Table 1) evidenced the absence of significant differences between this and the different independent variables proposed in the study. On the contrary, the path analysis shows that the PSU had a negative and significant influence on the self-esteem of the students, as evidenced by other studies [29,30,31,32]; while the Instagram use intensity had no effect on it. This last fact contrasts with other investigations [33,43], which suggest that participation in social networks is linked to elements such as insecurity, poor social skills or attributes.

Among the limitations presented by this study is the cross-sectional nature of the study, since it only represents that sample and at that time. A longitudinal study would be necessary to obtain data that could be assumed to be representative. In addition, the study has been carried out only with students in the specialty of physical education and by means of convenience sampling.

## 5. Conclusions

The study carried out shows that the self-esteem construct, as part of the person’s personality, is negatively affected by the PSU. This PSU is encouraged by socio-demographic factors such as youth, an inactive work situation, and participation in social networks such as Instagram. On the other hand, an intensive use of this social network does affect the PSU and the need to publish more photographs and the increase in the number of followers.

As it can be seen, the relationships between the three dimensions (PSU, Instagram, and Self-esteem) are clearly defined in the path analysis, where self-esteem decreases in relation to the increase in the PSU, and this, in turn, increases with Instagram use intensity. In this sense, training in the responsible use of technological devices at early ages plays a determinant role in the prevention of future cases of PSU. Furthermore, this training should be aimed at making subjects aware of the importance of their privacy and ensuring a coherent digital identity, understanding that the public exposure of personal facets may have negative consequences in the present and in the future.

The study has answered the four questions raised by providing very significant data that can offer a very interesting line of work. This line of work could be aimed at studying the attitudes developed by people who have a PSU and intensive use of Instagram, and propose specific actions for its prevention and treatment.

## Figures and Tables

**Table 1:** Means, standard deviations and differences between independent variables in different scales.

Variables	Instagram			PSU			Self-Esteem		
	M	SD	*p*	M	SD	*p*	M	SD	*p*
Gender									
Male	16.72	4.715	0.912	19.88	5.704	0.943	32.68	5.755	0.444
Female	16.78	4.884		19.92	5.613		32.20	6.076	
Age									
≤20	18.66	4.582	0.000	20.93	6.130	0.034	31.41	6.309	0.057
21–35	16.09	4.721		19.54	5.424		32.73	5.795	
Employment status									
Active	16.02	4.621	0.002	19.20	5.768	0.011	32.93	6.200	0.063
Inactive	17.54	4.903		20.66	5.415		31.80	5.634	

Note: *p* calculated through the T test; PSU = Problematic Smartphone Use; SD = Standard Deviation.

**Table 2:** Univariate data normality.

Dimension	Skewness	Kurtosis	Kolmogorov–Smirnov		
			K-S	*df*	*p*
Instagram Use Intensity	−0.108	−0.594	0.061	385	0.002
Problematic Smartphone Use	0.609	0.031	0.082	385	0.000
Self-esteem	−0.744	0.054	0.114	385	0.000

Note: *df* = degrees of freedom.

**Table 3:** Goodness of fit measure.

Fit Indices	Obtained Values	Criteria
χ^2^	10.44	
*df*	8	
χ^2^/*df*	1.305	≤3
GFI	0.993	≥0.90
RMSEA	0.028	<0.05
NFI	0.973	≥0.90
CFI	0.993	≥0.90
AGFI	0.969	≥0.90
SRMR	0.025	<0.08

Note: GFI = Goodness-of-Fit Index; RMSEA = Root Mean Squared Error of Approximation; NFI = Normalised Fit Index; CFI = Comparative Fit Index; AGFI = Adjusted Goodness-of-Fit Index; SRMR = Standardized Root Mean Square Residual.

**Table 4:** Parameter estimates of final model.

Associations between Variables	RW	SE	CR	*p*	SRW
Instagram ← Gender	1.034	0.523	1.976	0.048	0.104
Instagram ← Age	−2.658	0.632	−4.202	***	−0.242
Instagram ← Employment situation	0.687	0.523	1.313	0.189	0.071
Instagram → No. Photos	8.460	2.030	4.168	***	0.205
Instagram → No. Followers	92.338	16.954	5.446	***	0.268
Instagram → Self-esteem	0.083	0.069	11.199	0.230	0.067
PSU ← Gender	0.630	0.645	0.977	0.329	0.054
PSU ← Age	−1.073	0.769	−1.395	0.163	−0.084
PSU ← Employment situation	1.124	0.632	1.777	0.076	0.100
PSU ← No. Photos	−0.001	0.001	−0.518	0.605	−0.025
PSU ← No. Followers	0.000	0.000	−0.959	0.338	−0.045
PSU → Self-esteem	−0.333	0.059	−5.668	***	−0.316
Instagram ↔ PSU	0.172	0.028	6.154	***	0.492

Note: PSU = Problematic Smartphone Use; RW = regression weights; SE = standard error; CR = critical radio; SRW = standardized regression weights; *** *p* < 0.001; ←, → = relationship direction; ↔ = correlation.
